# The effect of online motivational interviewing on stress management in infertile women with PCOS: A randomized clinical trial

**DOI:** 10.1192/j.eurpsy.2021.2017

**Published:** 2021-08-13

**Authors:** F. Ansari, Z. Hamzehgardeshi, F. Elyasi, M. Moosazadeh, I. Ahmadi

**Affiliations:** 1 Master Student In Midwifery Counselling “student Research Committee, Mazandaran University Of Medical Sciences, Sari, Iran”, Mazandaran University of Medical Sciences, sari, Iran; 2 Sexual And Reproductive Health Research Center, Department Of Reproductive Health And Midwifery, School Of Nursing And Midwifery, Mazandaran University Of Medical Sciences, Sari, Iran, Mazandaran University of Medical Sciences, Sari, Iran; 3 Neurology, School Of Medicine Psychiatry And Behavioral Sciences Research Center Addiction Research Institutes Sari Imam Khomeini Hospital, Mazandaran University of Medical Sciences, sari, Iran; 4 Health Sciences Research Center Addiction Research Institutes, Mazandaran University of Medical Sciences, sari, Iran; 5 Department Of Obstetrics And Gynecology., School Of Medicine Sexual And Reproductive Health Research Center Sari Imam Khomeini Hospita, Mazandaran University of Medical Sciences, SARI, Iran

**Keywords:** Infertile women, polycystic ovary syndrome, motivational interview, Health-promoting behaviors, stress

## Abstract

**Introduction:**

Polycystic ovary syndrome (PCOS) is one of the most common endocrine disorders in women, which in addition to medical aspects also affects the dimensions of women’s mental health such as stress.

**Objectives:**

The present study was conducted to determine The effect of online motivational interviewing on stress management in infertile women with PCOS

**Methods:**

This randomized controlled clinical trial enrolled 60 Infertile Women with PCOS from the city of Sari-Iran in 2020. Participants were assigned to MI and control groups using block randomization. The intervention group received 5 weekly of MI online via WhatsApp. While the control group received only routine care. Stress management scores in these individuals were measured using health-promoting lifestyle profile II questionnaire (HPLP II) before and after the intervention. Then, the data were entered into the SPSS software, version 25 and were analyzed using descriptive statistics, chi-square test, t-test, and repeated measures analysis of variance.

**Results:**

No significant difference was observed between the two groups before the intervention mean The Stress management scores (p>0.05). After the intervention, mean (SD) of The Psychological Domains score was 22.5 (3.8) in the intervention group and 17.9 (4.1) in the control. The Stress management score was significantly upper in the intervention group compared to the control. (p<0.000). The effect size (1.1) was calculated. NNT (1.6) was calculated.

**Conclusions:**

According to the results and the effect of motivational interviewing is one of the effective methods to manage stress in infertile women with PCOS.

**Disclosure:**

No significant relationships.

